# mHealth Technologies for Palliative Care Patients at the Interface of In-Patient to Outpatient Care: Protocol of Feasibility Study Aiming to Early Predict Deterioration of Patient’s Health Status

**DOI:** 10.2196/resprot.7676

**Published:** 2017-08-16

**Authors:** Gudrun Theile, Vanessa Klaas, Gerhard Tröster, Matthias Guckenberger

**Affiliations:** ^1^ Clinic of Radiation-Oncology Competence Center Palliative Care University Hospital Zurich Zurich Switzerland; ^2^ Wearable Computing Laboratory Swiss Federal Institute of Technology Zurich Switzerland

**Keywords:** mobile apps, palliative care, pain, symptom assessment, hospitalization, aged

## Abstract

**Background:**

Palliative care patients are a particularly vulnerable population and one of the critical phases in patients’ trajectories is discharge from specialized in-patient palliative care into outpatient care, where availability of a palliative care infrastructure is highly variable. A relevant number of potentially avoidable readmissions and emergency visits of palliative patients is observed due to rapid exacerbation of symptoms indicating the need for a closer patient monitoring. In the last years, different mHealth technology applications have been evaluated in many different patient groups.

**Objective:**

The aim of our study is to test feasibility of a remote physical and social tracking system in palliative care patients.

**Methods:**

A feasibility study with explorative, descriptive study design, comprised of 3 work packages. From the wards of the Clinic of Radiation-Oncology at the University Hospital Zurich, including the specialized palliative care ward, 30 patients will be recruited and will receive a mobile phone and a tracking bracelet before discharge. The aim of work package A is to evaluate if severely ill patients accept to be equipped with a tracking bracelet and a mobile phone (by semiquantitative questionnaires and guideline interviews). Work package B evaluates the technical feasibility and quality of the acquired electronic health data. Work package C will demonstrate whether physical activity parameters, such as step count, sleep duration, social activity patterns like making calls, and vital signs (eg, heart rate) do correlate with subjective health data and can serve as indicator to early detect and predict changes in patients’ health status. Activity parameters will be extracted from the mobile phone’s and wristband’s sensor data using signal processing methods. Subjective health data is captured via electronic version of visual analog scale and Distress Thermometer as well as the European Organization for Research and Treatment of Cancer – Quality of Life Questionnaire C30 in paper version.

**Results:**

Enrollment began in February 2017. First study results will be reported in the middle of 2018.

**Conclusions:**

Our project will deliver relevant data on patients’ acceptance of activity and social tracking and test the correlation between subjective symptom assessment and objective activity in the vulnerable population of palliative care patients. The proposed study is meant to be preparatory work for an intervention study to test the effect of wireless monitoring of palliative care patients on symptom control and quality of life.

## Introduction

Severely ill patients and their caregivers face many challenges. Suffering from a life-threatening disease not only confronts people with deeply existential fears that demand complex coping strategies. It also means to be subdued to numerous diagnostic and therapeutic interventions, which sometimes themselves include relevant risks of side effects and complications. In the last months of their lives many palliative care patients are afflicted with different significant symptoms, such as pain, nausea, dyspnea, fatigue, and sleeplessness, just to name the most frequently occurring complaints [1-3].

One of the particular critical phases in patients’ trajectories is discharge from hospital care. After structured and multiprofessional care within the hospital environment, maybe even at a specialized Palliative Care Unit, the quality of care at home depends on many local factors that are difficult or impossible to be changed: patients` environment, including housing, social, and family situation, as well as availability and training of local professional health services. Due to different initiatives on the federal and cantonal level, the availability of ambulant specialized palliative care services in Switzerland is on the rise. Still, most patients discharged from hospital are lost to follow-ups by a palliative care specialist. As a consequence, many patients present for unplanned readmissions at hospitals and emergency departments [4]. Depending on different settings, between 17% and 50% of these (re)admissions and emergency visits (EV) of cancer patients are deemed avoidable [5-7]. Men, lung cancer patients, patients with low continuity of care, and those not attending (ambulant) palliative care have been shown to be particularly vulnerable [8,9]. Main causes for EV in cancer patients are uncontrolled symptoms, predominantly pain, nausea, vomiting, constipation, and dyspnea followed by complications associated with cancer treatment [10-12]. Currently, disease trajectories leading to EV are not well understood and this is not only true for cancer patients, but also applying to patients with life-limiting chronic diseases (eg, heart failure); continuity of care seems to also be one of the key factors in both disease spectra [13,14]. Thus, better coordination and communication between services, establishing transitional palliative care (based in hospital, collaborating with primary care physicians) are repeatedly named as potentially effective tools to diminish unplanned readmissions and EVs [2,4,9,15].

Due to a dramatic development of mHealth the use of apps and texting for patients’ support, but also wireless tracking of physical activity using mobile phones and wearable sensors has been expeditiously adopted in western societies in the last few years. In 2015, 63% of the US adult population have owned a mobile phone device and even in the age group >65-years old, mobile phones were owned by 27% [16]. A study conducted in patients from a department of Internal Medicine in Los Angeles in 2014 to 2015 showed that 91% of those patients owned a mobile phone, with 76% of these reporting having Internet capability. Mobile apps were used by 75% of patients and 32% of these even used health apps [17]. The use of mobile phones and mobile sensor devices in medical research is also growing fast: almost US $1 billion in grant money was invested in mHealth research in 2013 resulting in approximately 1000 web-of-science publications in 2013 [18]. Reasons for this tremendous hope on mHealth are their possibilities to obtain various objective patient-related parameters in a noninvasive, passive, continuous, and real-time fashion.

Meanwhile mHealth has been explored in many diseases and patient groups, for example, in cardiovascular diseases [19], mental disorders [20,21], obese people [22], hemodialysis patients [23], chronic pain [24], children with cerebral palsy [25], pulmonary rehabilitation [26], Parkinson’s disease [27], and stressed persons [28]. Also, the collection of patient subjective reported outcome using mHealth is already established in oncology and yet has been proven feasible in palliative care patients of different age groups [29-33]. In a study with 162 cancer patients in different disease stages (early to advanced) plus 20 healthy controls, Ferrioli et al. [34] could demonstrate a strong correlation between daily physical activity monitored by an accelerometer sensor and disease stage, functional status, fatigue, and quality of life (QoL) in cancer patients [34]. Patients were equipped with a mobile sensor that was attached to the anterior thigh using adhesive dressings (thus, being much more uncomfortable than current bracelet devices); nevertheless, 98% of patients judged the sensor as acceptable and wore it 7 days as proposed. Arguments based on the assumption the population of elderly people could not adapt to the technical progress have been successfully refuted by several studies [35-37]. Consequently, the international scientific palliative care community promotes the use of mHealth for quite a while, but up to now there has not been much experience gained and published [38].

The overall aim of our feasibility study is to advance transitional care and continuity of care of seriously ill patients by application of mHealth technologies. We assume that by monitoring patients` activity using mHealth technology, we will be able to predict a decline of the patients` health status in time: this could then trigger intensified care by palliative care professionals. The future goal of our research is a clinical interventional trial, which aims at reducing unplanned hospital readmissions and EV of ambulant palliative care patients by the use of mHealth. The feasibility study presented in this paper will evaluate and optimize patients’ acceptance of mHealth technologies in the palliative care setting of patients discharged from in-patient care. We aim to analyze the correlation between patient’s behavioral patterns and changes in crucial symptoms as well as elaborate algorithms for trend detection.

## Methods

### Aims of the Study

The first aim of this study is to evaluate palliative care patients’ acceptance with regard to the supply with a commercially available wireless physical activity tracking bracelet and a mobile phone in order to monitor objective health and behavioral data and to capture subjective symptom assessment. The second aim is the evaluation of correlations between the patient-specific activity patterns (physical, social activity, and vital signs) and the subjective patients’ ratings of pain, distress, and QoL in order to early detect changes in the patient-specific pattern of physical and social behavior, vital sign patterns, and the named symptom ratings.

### Characteristics of Participants

Patients will be consecutively recruited at the 2 wards of the clinic of radiation-oncology of the University Hospital Zurich (USZ). One ward is part of the Competence Center Palliative Care USZ and offers specialized palliative care for patients with life-threatening diseases from all clinics of the university hospital. Most of these patients do suffer from cancer, a minority from incurable heart failure, severe pulmonary affections, or neurologic diseases. Inclusion criteria are: patients with established diagnosis of metastatic cancer or other severe illness with limited life-expectancy (physicians guess <12 months, >8 weeks), Karnofsky Index ≥50%, scale of performance status developed by the Eastern Cooperative Oncology Group (ECOG) ≤2, aged >18 years. Eligible patients with interest of participation will have to pass a practical minitest concerning the handling of the devices (ie, simple operations on a mobile phone and handling of activity bracelet). Exclusion criteria are relevant cognitive impairment and insufficient knowledge of German language. Similar to related work [20,39] a number of 30 participants is intended to ensure relevant findings by capturing a representative cohort regarding sex, age, health status (Karnofsky Index/ECOG), life-limiting diseases, and different grades of pre-existing experience in the use of mobile phones or other electronic devices. Patients interested in the study will receive a leaflet with sound information on the aim of the study, a rough sketch of study procedure, and requirements with regard to participants (eg, wearing a tracking bracelet). Attached at this information leaflet is the informed consent. Moreover, we hand out a second leaflet with detailed information on the handling of the tracking bracelet, the app, and characteristics of data gathered by device and app.

### Study Design

For this feasibility study, we chose an explorative descriptive design observing palliative care patients discharged from hospital and equipped with physical activity tracker and mobile phone technology. The underlying assumption is that wireless tracking of physical and social activity as well as of vital sign data in the palliative care setting is accepted by patients, is feasible, and its pattern analysis allows the generation of objective information on the current health status in the vulnerable phase of leaving inpatient care. For specific study question, see Textbox 1.

**Figure 1:**
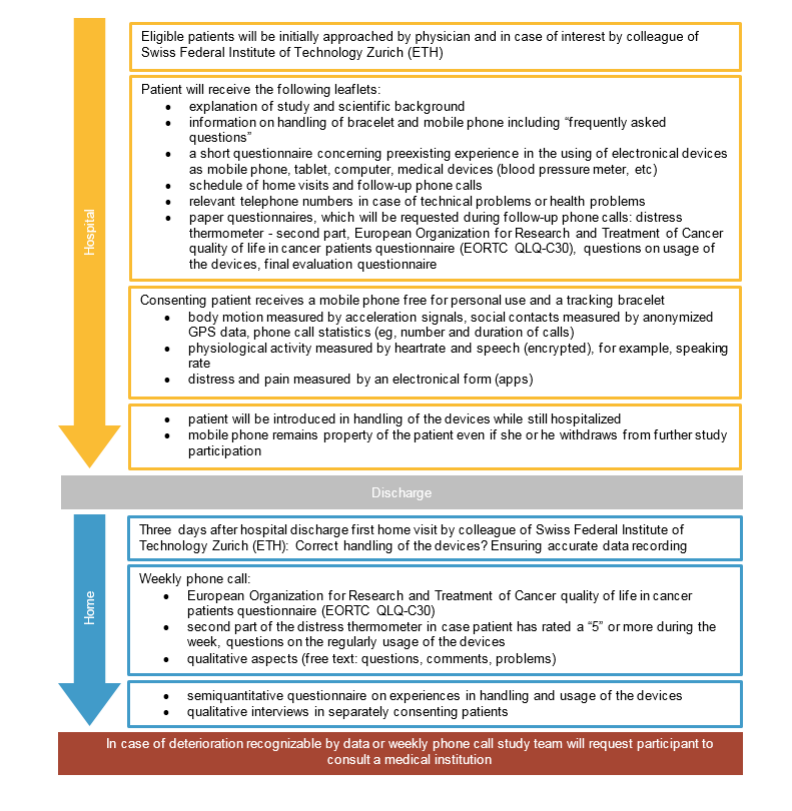
Study flow and explanation of different procedures.

Patients leaving inpatient care of the Clinic of Radiation-Oncology will be equipped with a mobile phone and tracking bracelets. We will use the Samsung Galaxy S5 mobile phone and it will capture body motion data (by acceleration signals), location (anonymized global positioning system [GPS] data), encrypted speech, and phone call statistics. Furthermore, in this study, a mobile phone questionnaire app will be installed, a visual pain scale, and the Distress Thermometer. Patients will be requested to answer these simple to handle questionnaire app twice a day. The tracking bracelet used in this feasibility study is provided by Biovotion company, it is worn at the upper arm, easy to put on, and it will capture heart rate, heart rate variability, blood oxygen, blood perfusion, skin temperature, stress (by galvanic skin response), and body motion data (by acceleration data). Figure 1 gives a detailed overview on study procedures.

**Specific questions of the feasibility study.**

- Are severely ill patients willing and able to join the study?
- Are severely ill and often elderly patients able to handle mHealth devices and mobile apps presenting visual scales?
- How can we support correct handling of the electronic devices to achieve good data quality?
- What is the patient selection criteria for the use of mHealth?
- Feasibility, acceptance, and technical functionality in the home care setting.
- Patient acceptance and feasibility over a longer follow-up period (maximum 12 months).
- How accurate is the data collected and transferred by the devices to the study center over the follow-up period?
- How accurate is data of physical and social activity trackers correlated with subjective reported outcomes by patients?
- Is it possible to detect behavioral changes, especially within person, with gathered data?
- Is it possible to extend a patient-specific model with a general model?

**Work package A: patient acceptance of mHealth.**

- Quantitative (descriptive statistics) and qualitative evaluation of usage, acceptance, and potential problems during follow-up (maximum 12 weeks) within home visits and/or weekly telephone interviews.
- Definition of criteria for successful application (patients’ selection, instructive approaches).
- Final evaluation of usage and potential problems by semiquantitative questionnaire at the end of follow-up, guideline interviews of some of the patients, and of the involved personnel.
- Descriptive evaluation of problems indicated in the second part of the Distress Thermometer (if applicable).
- As nonresponders will be defined those patients, who are approached to participate but who decline from participation. We will capture demographic date of nonresponders, including main diagnosis if these patients gave general informed consent to the University Hospital for usage of their data from electronic records for research purposes. Additionally, we will ask nonresponders to document their reason for decline in a short paper form; they have to consent to this procedure by giving as a signature. As drop-outs will be defined those patients, who gave consent for participation, but drop out during follow-up for other reason than death.

### Planned Data Collection and Analysis

With regard to analysis, the study is composed of 3 work packages: A, B, and C. Work packages A and B enclose interims analysis; that way preliminary evaluation results can be implemented during patient recruitment phase aiming continuously to improve feasibility and data quality. In Textboxes 2-4, we describe the different work packages.

### Work Package B – Technological Feasibility of Behavior Tracking and Monitoring of Health Status

Work package B evaluates whether physical activity parameters, such as step count, sleep duration, social activity patterns like making calls, going out to meet people, and vital signs (eg, heart rate) can be technically measured over sufficiently long periods and whether this data correlate with changes in the health status of patients. Health status will be assessed in terms of the following parameters in Textbox 3.

### Work Package C - Data Analysis, Correlation, and Trend Detection

The recorded data of the bracelet and mobile phone are uploaded to a secured webserver of the Swiss Federal Institute of Technology once per day to allow remote access to the data (only for the conductors of the study) in order to ensure accurate recording and for evaluation purposes. We chose the Biovotion bracelet, because it gives us the possibility to avoid data storage in a cloud. Additionally, it is an officially recognized medical device. Privacy sensitive data as real location (GPS) is anonymized, speech is encrypted, names and phone numbers of contacts are not recorded. All patient sensitive data is encoded and accessed only by people determined by the principal investigator. The local Ethics Committee (Kantonale Ethikkommission Zürich) has already approved the study and agreed to detailed specifications on data security we gave to them. Textbox 4 gives an overview on planned data collection and analysis.

**Work package B: subjective parameters.**

- Pain measured by an electronic form of a visual analogue scale (rating scale 0-10)
- Distress measured by an electronic form of the National Comprehensive Cancer Network Distress Thermometer [40] part 1 (rating scale 0-10); part 2 will be administered in weekly phone calls if ratings in the distress app were 5 or higher during the previous week.
- Quality of life: European Organization for Research and Treatment of Cancer Quality of Life Questionnaire-C30 [41,42].

**Planned data analysis.**

- Evaluation of the correlation (eg, the Pearson coefficient, statistical tests) between the patient-specific activity patterns (physical, social activity, and vital signs) and the ratings of the National Comprehensive Cancer Network Distress Thermometer, the pain visual analogue scale, and the European Organization for Research and Treatment of Cancer Quality of Life Questionnaire-C30.
- Quantitative ranking of the activity parameters regarding their relevance to describe the patient’s pain and distress status using statistics and machine learning (eg, minimum redundancy maximum relevance).
- Development of models to detect the patient’s current pain and distress level based on analysis of behavioral patterns and trend detection as well as knowledge-driven approaches.
- Evaluate the potential of the proposed activity tracking system to predict the health status of patients by assessing the accuracy of the prediction model using the patient questionnaire data as reference.
- Record the interventions needed based on the early detection of activity changes seen, including (emergency) hospital readmission.
- Application of adaptively train semisupervised classifiers, such as support vector machines, hidden Markov models, and clustering methods for trend detection and detection of behavioral changes, especially within person.
- Application of knowledge-driven approaches in order to compensate missing data to generate general model out of patient-specific model.

## Results

Enrollment began in February 2017. Data collection will be completed in January 2018, first results will be reported in the middle of the year.

## Discussion

Continuous symptom assessment and critical review of symptoms by patient and doctor have been defined as a mainstay of effective ambulant palliative care [39]. Yet, in many European countries primary care practices are rarified [43], reimbursement of home visits are insufficient [44], and despite different movements to embed palliative care in the public health care system ambulatory palliative care institutions are still lacking. Our study aims to detect early health status changes with the help of mHealth technology in order to improve health care for ambulatory palliative care patients and avoid unplanned or emergency hospital readmissions. As to our knowledge with regard to this special patient population, no data on correlation of subjective symptom burden and objective activity parameters do exist until now, this project will deliver highly novel data. Not only on aspects of feasibility and patients` acceptance, but particularly on the proof (or the rejection) of the presumed existence of a correlation between subjective and objective data. Due to the fact, we collect a broad range of activity data reaching form blood oxygen, perfusion, skin temperature and heart beat to call statistics, and voice modulation, we will be able to identify parameters that correlate best with the patient’s subjective symptom burden. This is important for the future development of effective mHealth systems supporting remote symptom management by wearables combined with proactive care in a patient group that is in constant danger of a rapid and/or unexpected deterioration in health status. With the help of advanced data analysis algorithm we will be able not only to detect subtle changes in health status sometimes initially unnoticed by the patient and his/her family, but relevant precursor of possible symptom exacerbations. This approach will furthermore allow for even predicting these sometimes very finely graduated changes in time; thus, enabling professionals with data access to initiate effective intervention in time. We are aware of the fact that equipment of palliative patients with tracking devices requires sensitivity and the goal of palliative care remains to provide the best QoL possible, not only with respect to medical, but also to psychosocial and spiritual needs. This project therefore aims to supplement and assist existing palliative care structures and resources to build-up effective home care programs, which provide comprehensive and coordinated care closely adapted to the patient individual needs.

## Abbreviations

ECOG: scale of performance status developed by the Eastern Cooperative Oncology Group
EV: emergency visits
GPS: global positioning system
QoL: quality of life
USZ: University Hospital Zurich

## Appendix

Multimedia Appendix 1 Funding report.

Multimedia Appendix 2 CONSORT eHealth Form.
