# Supplementary material for: mHealth Technologies for Palliative Care Patients at the Interface of In-Patient to Outpatient Care: Protocol of Feasibility Study Aiming to Early Predict Deterioration of Patient’s Health Status
Source: JMIR Res Protoc. 2017 Aug 16;6(8):e142. doi: 10.2196/resprot.7676 (PMC5577455; doi:10.2196/resprot.7676)
Supplement: Multimedia Appendix 1 [file resprot_v6i8e142_app1.pdf]

SAMWASSM
Schweizerische Akademie der Medizinischen Wissenschaften
Académie Suisse des Sciences Médicales
Accademia Svizzera delle Scienze Mediche
Swiss Academy of Medical Sciences

Frau
Dr. Gudrun Theile
UniversitätsSpital Zürich
Radio-Onkologie/ Kompetenzzentrum
Palliative Care
Rämistrasse 100
8091 Zürich

Bern, 10. November 2016

Förderprogramm «Forschung in Palliative Care»:
Ihr Gesuch: PC 16/16

Sehr geehrte Frau Dr. Theile

An ihrer Sitzung vom 14. September 2016 hatte die Expertenkommission des Förderprogramms «Forschung in Palliative Care» 25 teilweise hochkarätige Gesuche zu beurteilen. Die beantragten Projektbeiträge beliefen sich insgesamt auf über 4 Mio. Franken; der Kommission standen jedoch lediglich CHF 1 Mio. Franken zur Verfügung. In einem sorgfältigen Evaluationsprozess wählte die Kommission 8 qualitativ hochstehende Gesuche aus, die aus ihrer Sicht den reglementarisch festgehaltenen Förderungskriterien des Förderprogramms am ehesten entsprechen.

Wir freuen uns, Ihnen mitzuteilen, dass der Stiftungsrat der **Bangerter-Stiftung** auf Empfehlung der Expertenkommission beschlossen hat, Ihr Projekt «Mobile health technologies for palliative care patients at the interface of in-patient to out-patient care: A feasibility study to predict deterioration of patient's health status and aiming to prevent unplanned hospital re-admissions» mit einem Beitrag von CHF 132863.– zu unterstützen. Bitte geben Sie uns Ihre detaillierten Kontoangaben (Kontonummer, Adresse und wenn möglich Referenznummer) an, so dass die Stiftung die erste Tranche in den nächsten Wochen überweisen kann.

Bei einjährigen Projekten erwarten wir nach Projektabschluss einen wissenschaftlichen Bericht und eine Schlussabrechnung, bei mehrjährigen Projekten jährliche Zwischenberichte. Die Jahrestranchen werden jeweils nach Erhalt und Prüfung des Zwischenberichtes ausbezahlt. Wir möchten Sie daran erinnern, dass die Unterstützung durch das Förderprogramm «Forschung in Palliative Care» auf allen Publikationen resp. Postern und Präsentationen wie im Reglement vorgeschrieben ausgewiesen werden muss.

Wir wünschen Ihnen viel Erfolg bei der Durchführung Ihres Projektes und grüssen Sie freundlich.

Dr. Tapernoux

Dr. Myriam Tapernoux
Ressort Forschung

Kopie: Prof. Peter Meier-Abt, Präsident Expertenkommision; Gabriela Kyburz, Geschäftsführerin Bangerter-Stiftung

SAMWASSM, Haus der Akademien, Laupenstrasse 7, CH-3001 Bern
Telefon +41 31 306 92 70, mail@samw.ch, www.samw.ch

Mitglied der
a+ akademien der wissenschaften schweiz
